# The Changing Landscape of Pulmonary Arterial Hypertension in the Adult with Congenital Heart Disease

**DOI:** 10.3390/jcm6040040

**Published:** 2017-03-30

**Authors:** Alexandra C. van Dissel, Barbara J. M. Mulder, Berto J. Bouma

**Affiliations:** 1 Department of Cardiology, Academic Medical Center, Meibergdreef 9, 1105 AZ Amsterdam, The Netherlands; a.c.vandissel@amc.uva.nl (A.C.v.D.); b.j.mulder@amc.uva.nl (B.J.M.M.); 2 Netherlands Heart Institute, Moreelsepark 1, 3511 EP Utrecht, The Netherlands

**Keywords:** pulmonary arterial hypertension, pulmonary hypertension, congenital heart disease, Eisenmenger syndrome, therapy

## Abstract

Pulmonary arterial hypertension associated with congenital heart disease (PAH-CHD) is a common type of pulmonary arterial hypertension (PAH) and a frequent complication of congenital heart disease (CHD). PAH-CHD represents a heterogeneous patient population and it is important to distinguish between the underlying cardiac defects considering the prognostic and therapeutic implications. Improved interventional techniques have enabled repair or palliation of most cardiac defects, though a substantial number of patients remain at high risk for PAH after closure. Traditionally, the treatment and management of PAH-CHD patients has been limited to palliative and supportive care, and based on expert opinion rather than clinical trials. Recently, however, the availability of advanced PAH-specific treatment has opened up a new field for the clinical management of this condition. Nevertheless, there is limited evidence on the optimal therapeutic approach for PAH-CHD. Herein, we discuss the current and novel therapeutic options for PAH-CHD as well as highlight several challenges in the clinical management at present.

## 1. Introduction

Any congenital heart disease (CHD) with intra- or extracardiac shunting that allows for persistent exposure to increased pressure and volume overload of the pulmonary circulation may lead to the development of pulmonary arterial hypertension (PAH). PAH may develop at any stage of the disease and when it does, it is characterized by poor exercise tolerance and quality-of-life as well as high morbidity and mortality [1]. Despite similarities in terms of pulmonary histology, pulmonary arterial hypertension associated with congenital heart disease (PAH-CHD) differs distinctly from other PAH etiologies with regards to cardiac anatomy, pathophysiology, and clinical outcome, as it represents a heterogeneous patient population with myriad underlying cardiac defects, previous interventions, and associated comorbidities. During the past years, there have been notable advances in novel PAH-specific therapies, with a major impact on the clinical management of the disease. However, limited evidence is available on the use of these advances therapies for PAH-CHD. The anticipated growing number and complexity of adult congenital heart disease (ACHD) survivors [2] raises further practical concerns as many CHD patients are at increased risk for developing PAH. Consequently, PAH-CHD patients and adult cardiologists face distinct challenges in clinical practice. Herein, we discuss the current and novel insights for treatment of PAH-CHD, with particular focus on certain challenges commonly encountered in clinical practice when caring for these patients.

## 2. Epidemiology and Genetics

**Figure 1:**
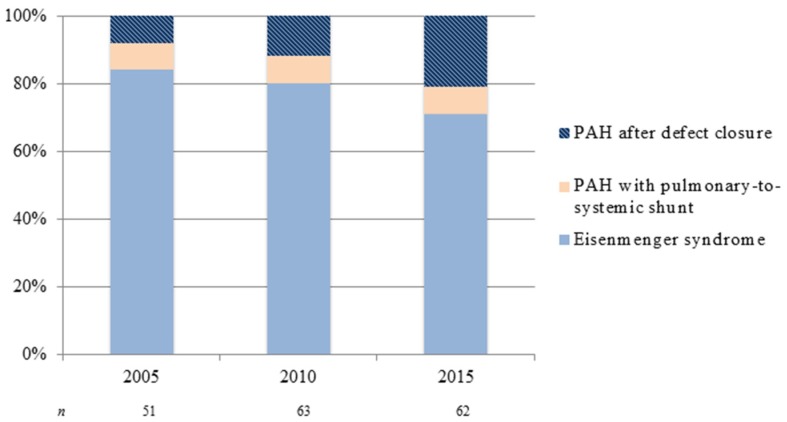
Changing subgroup distribution of pulmonary arterial hypertension in adult congenital heart disease: patients on treatment at two congenital heart disease (CHD) designated centers in the Netherlands from 2005 to 2015. PAH: pulmonary arterial hypertension.

The demographics of CHD and subsequently PAH-CHD are changing. Over the last decades, major advances in pediatric cardiovascular medicine and surgery have led to a marked increase in survival of CHD patients—more than 90% now reach adulthood [3]. The remarkable improvement in survival has led to a continuously growing number of ACHD patients, in particular those with more complex CHD. The estimated incidence of CHD is 7–9 per 1000 live births [4]. Approximately 5%–10% of CHD patients will go on to develop PAH, depending on location, size (i.e., both anatomical and degree of shunting), and type of cardiac defect [5,6,7]. The development and severity of PAH may accompany a variety of forms of CHD. For instance, the risk for developing Eisenmenger syndrome (ES)—the end-stage form of PAH-CHD—appears to be determined by the size of the initial systemic-to-pulmonary shunt and the volume of pulmonary blood flow, with larger shunts having increased risk. In addition, the type of cardiac defect is important, as only a small percentage of patients with unrepaired atrial septal defects develop ES, compared to higher percentages of patients with unrepaired ventricular septal defects and complete atrioventricular septal defects [5,8]. Despite improved pediatric CHD care—likely preventing the development of pulmonary vascular disease—the overall number of adult patients with PAH-CHD nowadays seen in specialist centers appears to increase. The seven UK designated PAH centers reported a prevalence of PAH-CHD (30.2% of all PAH) equivalent to that of idiopathic PAH (33.6%) [9], much higher than previous reports, though this probably reflects raised awareness, closer collaboration between PAH and CHD centers and better referral liaisons. Also, there seems to be a shift in PAH-CHD subgroup distribution: as a result of early diagnosis and repair of CHD, the prevalence of Eisenmenger syndrome has declined [10]—though not negligible—whereas the number of adult patients with PAH after defect closure appears to increase, as is illustrated in Figure 1.

Interestingly, some CHD patients are at higher risk for developing PAH and with rapid disease progression despite similar underlying cardiac defects. Aside from environmental factors, genetic predisposition may play an important role in the phenotypic variability of PAH in CHD. It may be useful to screen for PAH gene mutations in case of discrepancy between severity of PAH and CHD. Multiple mutations have been identified in hereditary PAH. Some of which, like the bone morphogenetic protein receptor type 2 mutation, were also observed in PAH-CHD patients, though considerably less than in hereditary or idiopathic PAH patients (6% vs. 50% and 26%, respectively [11]. Research is required into understanding the genetic impact on the natural evolution of PAH disease, both in CHD and other types of the disease.

## 3. Classification of PAH

Pulmonary hypertension (PH) refers to diseases with increased pulmonary pressures, defined as mean pulmonary arterial pressure (PAP) ≥ 25mmHg at rest. PH is not synonymous with PAH, which specifies either obstructive or vasoconstrictive PH characterized by elevated pulmonary vascular resistance (PVR) and normal pulmonary venous pressure [12]. The clinical classification of PH describes five groups: (i) PAH, (ii) PH due to left heart disease, (iii) PH attributable to lung disease and/or hypoxemia, (iv) chronic thromboembolic PH and other pulmonary artery obstructions, and (v) PH with unclear and/or multifactorial mechanisms [13]. Group (i) PAH is further classified as idiopathic, heritable, drugs and toxin induced, and associated with either connective tissue disease, human immunodeficiency virus infection, portal hypertension, congenital heart disease, or schistosomiasis. Current guidelines have acknowledged the wide spectrum of PAH-CHD and four main groups are listed under PAH-CHD according to clinical phenotypes: (i) ES, (ii) PAH associated with prevalent systemic-to-pulmonary shunt lesions, (iii) PAH with small or coincidental cardiac defects, and (iv) PAH after defect closure (either persisting or recurring) [13,14,15] (Table 1). A recent update of the guidelines [13] also recognizes PH in the setting of congenital left heart inflow or outflow obstructive lesions, congenital pulmonary artery stenosis, and congenital cardiomyopathies, but these types do not belong to group (i) (PAH) and are included in other PH groups (ii), (iv), and (v) of the general classification.

PAH-CHD is distinctly different from other PAH etiologies regarding underlying cause and clinical outcome. In general, PAH-CHD has a slower disease progression and better life expectancy compared to other PAH etiologies [16]. Survival of ES patients is limited, even though the individual clinical course can be quite variable with some surviving into their 60s and beyond [17,18,19]. Of note, a recent study on the different clinical subgroups of PAH-CHD suggested that patients with ES or prevalent systemic-to-pulmonary shunts have a better survival compared to those with PAH after closed defect or with small defects [20]. Improved survival may result from preservation of the right ventricular (RV) function by prevention of remodeling during infancy—the ES heart is more like the fetal heart than the normal adult heart, and remains hypertrophied [21,22]—and the ability to relieve the RV and maintain cardiac output by shunting, albeit at the expense of cyanosis. These findings reinforce the need to delineate the underlying cardiac anatomy, pathophysiology, and severity of PAH in every individual patient.

## 4. Clinical Evaluation of PAH-CHD

As indicated in Table 1, the clinical presentation of PAH-CHD in adult patients varies depending upon the underlying cardiac defect and degree and direction of shunting. Clinical signs such as marked exercise intolerance with dyspnea and fatigue may raise the suspicion for PAH. ES represent the end-stage form of PAH-CHD, characterized by an initial systemic-to-pulmonary shunt leading to progressive PAH with ultimate reversal or bidirectional shunting and development of cyanosis. Cyanosis causes ES patients to be highly symptomatic with poor quality-of-life and severe functional limitation [23]. As ES is a multisystem disorder, patients may also present with an array of potentially life-threatening complications including hyperviscosity, abnormal hemostasis, and arrhythmias. Prognosis is among the worst in the ACHD population [24], though apparently better than PAH with small or closed defects [20].

**Figure 2:**
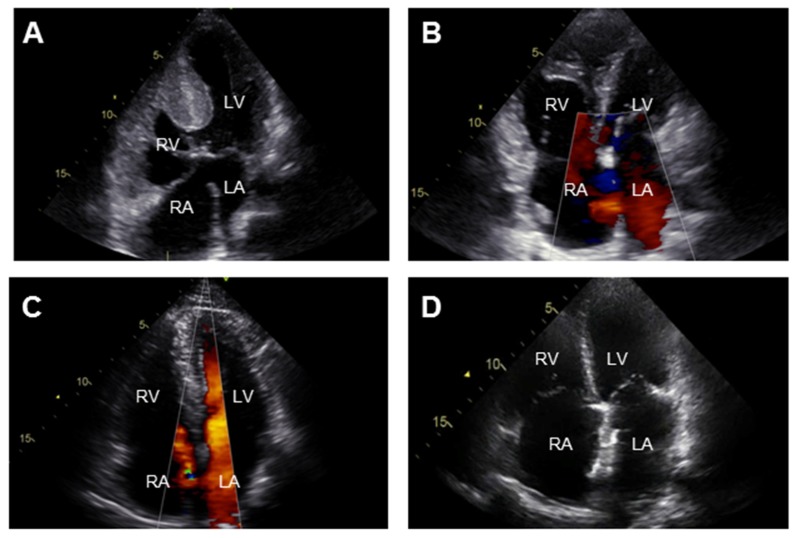
Transthoracic echocardiographic (apical four chamber view) images of the 4 clinical subgroups in pulmonary arterial hypertension associated with congenital heart disease (PAH-CHD): (**A**) Eisenmenger syndrome: atrioventricular septal defect; (**B**) PAH associated with prevalent systemic-to-pulmonary shunt lesion: atrial septal defect with left-to-right shunt; (**C**) PAH associated with small or coincidental cardiac defect: secundum atrial septal defect; (**D**) PAH after defect closure: closed atrial septal defect. LA = left atrium; LV = left ventricle; RA = right atrium; RV = right ventricle.

Transthoracic echocardiography is the most commonly used diagnostic test in the initial evaluation on PAH-CHD to determine the underlying cardiac defect, image the effects of PAH on the heart and provide an estimate of the PAP with continuous wave Doppler measurements (Figure 2). Additional imaging by cardiac magnetic resonance or computerized tomographic is indicated in case of elevated PVR in the absence of a detectable shunt on echocardiography and allows for assessment of cardiac anatomy and RV function. Right heart catheterization should be considered when non-invasive measurements are not conclusive or to support decisions on shunt correction. Laboratory testing is not useful in diagnosing PAH, but is required to identify associated disorders like thyroid disease and iron deficiency as well as provide information on disease severity and end-organ damage (with circulating biomarkers such as brain-natriuretic peptide, N-terminal pro brain natriuretic peptide (NT-proBNP) [25,26] and cystatin C [27]).

## 5. General Management

### 5.1. Expert Centers

International consensus guidelines recommend that PAH-CHD patients should undergo periodic assessment at specialized centers for both PAH and CHD [13,15]. A comprehensive understanding of the initial CHD diagnosis, previous interventions, and current anatomical and pathophysiologic features is crucial, though can reasonably at times exceed the expertise of physicians not specifically trained in this field. A Canadian study demonstrated improved survival among ACHD patients after the establishment of larger specialized centers nationwide [28]. Moreover, recent data from the German national registry for CHD suggest that ES patients treated at large specialized centers have a better outcome than those at follow-up in non-specialized centers, chiefly mediated by more frequent use of advanced therapies [29].

### 5.2. Supportive Care

Several supportive measures should be considered in management of PAH-CHD. PAH-CHD patients, especially those with ES, are at particular risk for hemodynamic complications during general anesthesia and surgery. For ES patients, a multidisciplinary approach is imperative and surgery should preferably be performed only at centers with expertise in the management of such patients, or in case of emergent or urgent situations, caregivers should seek consultation with expert centers throughout care. Pregnancy is still associated with a significant risk of both maternal and fetal mortality even though recent reports show better outcome than previously described as specialized care during pregnancy and delivery is now available [30,31]. However, given the severity and the high rate of complications associated with pregnancy, most PAH-CHD patients should still be advised against pregnancy. Appropriate counselling and care on contraceptive use should be part of the routine management of women of reproductive age. In view of the interaction with progesterone-based compounds, patients on treatment with endothelin-receptor antagonists must be advised to use dual contraception. Estrogen-containing compounds should be avoided due to the increased risk of thrombosis [32]. Women with PAH-CHD who become pregnant should receive individualized counselling by a cardiologist or obstetrician with expertise in the management of PAH-CHD. In case of severe PAH-CHD, the possibility for early pregnancy termination should be discussed, as this may be life-saving for the mother. For those who choose to continue with the pregnancy, regular consultation with a multidisciplinary team with appropriate expertise in CHD, PAH, and anesthesia is required. Iron deficiency is also common among PAH-CHD patients and iron replacement therapy has been shown to improve exercise tolerance and quality-of-life [33]. Cyanotic patients should, in fact, be screened for iron deficiency and provided with iron supplements accordingly [9]. Further, patients are advised to avoid strenuous exercise given the risk of systemic vasodilatation in the context of markedly reduced pulmonary vasodilator reserve, but there is increasing evidence for favorable effects of exercise training in CHD patients in general [23,34], and especially among ES patients [35,36]. Prevention of dehydration is also essential for these patients, who depend on adequate preload to maintain cardiac output and have a tendency for hyperviscosity. The use of supplemental oxygen therapy remains dubious: it may improve symptoms but has not been shown to modify survival [37]. In ES patients, the abnormal hemostasis—paradoxical state of both an increased risk for bleeding and increased risk for thrombosis—represents a complex issue [17,38]. So far, limited data on the efficacy and safety of anticoagulants are available to guide use. Guidelines discourage the routine use in ES patients and state that anticoagulants should be considered in case of atrial fibrillation and pulmonary artery thrombosis, in the absence of major bleeding [15]. Heart-lung or lung transplantation are alternative treatment options for ES with poor prognosis [39], though limited by the shortage of available donor organs and delayed listing for transplantation because of the reasonably good survival prospects of patients with ES treated medically compared with those suffering from idiopathic PAH.

## 6. Advanced PAH-Specific Therapies

After recent successes in clinical trials, advanced therapy is now widely used for the treatment of PAH. Most pivotal trials have included a small number of patients with closed defects—because of the similarities with idiopathic PAH—and were underpowered for formal subgroup analyses, thereby limiting the generalizability to the overall PAH-CHD population. Nevertheless, increasing evidence from observational studies and several randomized controlled trials in mainly ES support the benefit of pulmonary vasodilator therapies in PAH-CHD. Table 2 provides an overview of double-blind, randomized, placebo-controlled trials (RCTs) including PAH-CHD patients. Though none of these trials have been powered or designed to assess the effects on mortality in PAH-CHD, three retrospective analyses report better survival among ES patients treated with PAH-specific therapies compared to treatment naïve patients [29,40,41].

**Figure 3:**
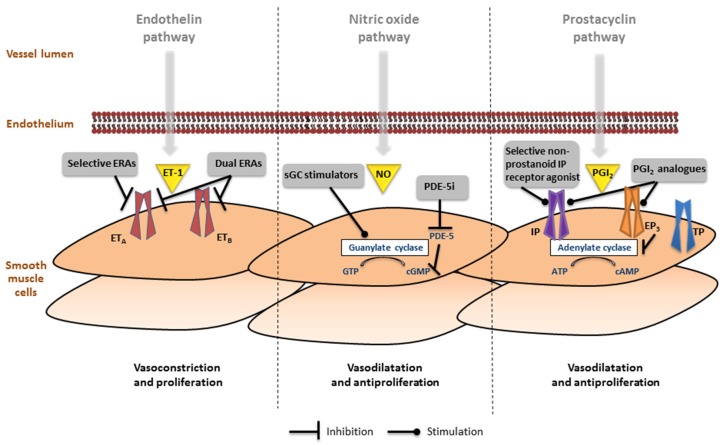
Involvement of the endothelin, nitric oxide (NO) and prostacyclin pathways in the pathogenesis of pulmonary arterial hypertension. (**Left**) In the endothelin pathway the effects of endothelin (ET)-1 are mediated via the ET_A_ and ET_B_ receptors in the smooth muscle cells. Receptor binding leads to mobilization of Ca^2+^ resulting in vasoconstriction and proliferation. Selective and dual endothelin receptor antagonists (ERAs) inhibit this pathway; (**Middle**) The nitric oxide pathway involves the production of cyclic guanosine monophosphate (cGMP), which leads to inhibition of Ca^2+^ entry, resulting in vasodilation and antiproliferation. Soluble guanylate cyclase (sGC) stimulators and phosphodiesterase type 5 inhibitors (PDE-5i) activate this pathway; (**Right**) In the prostacyclin (PGI_2_) pathway, prostanoid receptors IP, EP_3_, and TP regulate vessel tone (other prostanoid target receptors are not functionally expressed in the pulmonary artery). Selective non-prostanoid IP receptor agonists and PGI_2_ analogues bind to the IP receptor, which induces adenylate cyclase activity, cyclic adenosine monophosphate (cAMP) production, resulting in decreased Ca^2+^ and leads to vasodilation and antiproliferation. Some PGI_2_ analogues also bind to EP3 receptor leading to a decrease in cAMP, which blocks vasodilation.

To date, three major pathways have been identified in the pathophysiology of PAH and serve as target for PAH-specific treatment: (i) endothelin pathway, (ii) nitric-oxide pathway, and (iii) prostacyclin pathway (Figure 3).

### 6.1. Endothelin Pathway

Endothelin receptor antagonists (ERAs) include bosentan, macitentan (both dual ERAs; affect the endothelin A and B receptors), and ambrisentan (selective ERA; binds to endothelin A receptor). Currently, the strongest evidence for the use of ERAs in PAH-CHD is from the Bosentan Randomized Trial of Endothelin Antagonist Therapy-5 (BREATHE-5) and its 40-week open-label extension study. In this RCT with 54 ES patients, bosentan significantly improved exercise capacity, hemodynamics, and functional class [55,59], independently of the location of the septal defect and without compromising oxygen saturations [60]. Favorable long-term results from several studies have thereafter supported its use in PAH-CHD patients, especially ES [61,62,63].

Evidence supporting the use of ambrisentan in PAH-CHD is limited to one uncontrolled, single-center study with 17 ES patients [64] reporting an increased exercise capacity after 6 months. Results from subgroup analysis of a larger, retrospective study are disputable, as the majority of patients discontinued therapy due to side effects [65].

Macitentan has been granted approval based on the Study with an Endothelin Receptor Antagonist in Pulmonary Arterial Hypertension to Improve Clinical Outcome (SERAPHIN) trial, a RCT including 62 PAH-CHD patients with closed defects (8% of the study population) demonstrating a reduced morbity and mortality with macitentan [50]. The results from the Macitentan in Eisenmenger Syndrome to Restore Exercise Capacity (MAESTRO) trial in which 220 ES patients were randomized to assess the effects of macitentan on exercise capacity compared to placebo are expected this year (clinicaltrials.gov NCT01743001).

Currently, no studies on direct comparison have been performed to assess the superiority of one ERA over the other. Interestingly, however, a recent trial in which 40 PAH-CHD patients were switched from bosentan to macitentan, showed improved World Health Organization (WHO) functional class, NT-proBNP levels, and echocardiographic RV measures but noted no difference in events such as syncope and hospitalization for heart failure [66].

### 6.2. Nitric Oxide Pathway

To date, fewer studies on phosphodiesterase-5 inhibitors (PDE-5i), such as sildenafil and tadalafil, have been conducted in PAH-CHD compared to ERAs. One small randomized trial [56] with sildenafil demonstrated functional and hemodynamic favorable effects in 10 ES patients, and similar results were observed in uncontrolled series [67,68,69]. In the Sildenafil Use in Pulmonary Arterial Hypertension (SUPER-1) trial including 18 PAH-CHD subjects with closed defects (6% of the total study population), sildenafil treatment for 12 weeks led to an increase in exercise capacity and improvement in hemodynamics [47].

Similar favorable results of tadalafil have been reported in subgroup analyses of the Pulmonary Arterial Hypertension and Response to Tadalafil (PHIRST) trial [44], a preliminary study of 16 ES patients [70], and a crossover RCT in 28 ES patients [58].

A novel class of drugs targeting the nitric-oxide (NO) pathway are the soluble guanylate cyclase (sGC) stimulators, with riociguat as its first approved drug. The Pulmonary Arterial Hypertension Soluble Guanylate Cyclase–Stimulator Trial 1 (PATENT-1) included 35 PAH-CHD patients with closed defects (8% of the study population), and evaluated riociguat in treatment-naïve patients and patients on background therapy [45]. After 12 weeks, improvement in exercise capacity, hemodynamics, NT-proBNP, WHO class, and time-to-clinical worsening was found in the treatment group compared to the placebo group.

Despite the lack of supportive data, the efficacy and safety of sildenafil, tadalafil and riociguat seem comparable, though tadalafil does have the advantage of a once-daily dose intake compared to a three-time daily dose of sildenafil and riociguat. Also, riociguat requires a two weekly dose uptitration, whereas sildenafil and tadalafil do not require an uptitration phase.

### 6.3. Prostacyclin Pathway

Intravenous prostacyclin analogues were the first pulmonary vasodilators. In several small, open-label studies, intravenous epoprostenol led to favorable effects on functional class, hemodynamics and exercise capacity in PAH-CHD patients [71,72,73], but the applicability and safety of long-term intravenous administration with its associated risk of paradoxical embolism and line sepsis raises concerns. Thus, intravenous prostacyclins are generally not first-line therapy in PAH-CHD and have been confined to markedly functionally limited patients.

In a randomized trial with subcutaneous treprostinil [42], a subgroup of 109 PAH-CHD patients with closed defects or ES (23% of the total study population) were enrolled, and the beneficial effects on exercise capacity seemed not to differ from that observed in idiopathic PAH patients. In a prospective study with inhaled iloprost in 13 ES patients [74], 24 weeks of therapy led to improvements in exercise capacity and quality-of-life, though hemodynamics did not improve.

In general, oral therapies are preferred in PAH-CHD patients as opposed to intravenous drug given its potential risks of paradoxical embolism and infectious complications or inhaled drugs requiring frequent utilization. Recently, selexipag, the first oral selective non-prostanoid IP-receptor agonist, was approved for the treatment of PAH after the Prostacyclin Receptor Agonist In Pulmonary Arterial Hypertension Study (GRIPHON) trial [54]. In the GRIPHON trial, 1156 patients, either treatment-naïve or on background therapy including 110 PAH-CHD patients with closed defects, were randomized to receive placebo or selexipag in individualized doses. The risk for the composite end point of death or PAH-related complications was significantly lower with selexipag, and showed similar efficacy among the different baseline treatment groups and variable dose regimens. Selexipag definitely holds promise to expand treatment options by targeting a previously underutilized pathway, but its use in the context of other PAH-CHD patients with residual defects remains to be explored.

### 6.4. Combination Therapy

Over the last years, the experience with combination therapy—using two or more classes of drugs simultaneously—in PAH is increasing. Similar to treatment of systemic hypertension and heart failure, the rationale is to evoke additional and synergic therapeutic effects by acting on separate pathways at once. The general approach in PAH has been to start combination therapy when patients deteriorate or have suboptimal response to monotherapy. A recent meta-analysis of 18 RCTs including 4162 PAH patients showed that combination therapy reduced the risk of clinical worsening and was associated with improved functional status, exercise tolerance, and hemodynamics compared to monotherapy [75]. Despite this, reports from the REVEAL registry showed that 34.9% of PAH patients were receiving monotherapy at time of death [76]. Combination therapy may be applied sequentially or upfront [13].

Several RCTs provide evidence for favorable effects of sequential combination therapy in PAH [54,77,78,79,80], though limited data on use in PAH-CHD are available. D’Alto et al. suggested that sildenafil in addition to background bosentan significantly improved clinical status, exercise capacity and hemodynamics in 32 PAH-CHD patients with clinical worsening on bosentan monotherapy [81], while another study failed to show a benefit of addition of sildenafil to bosentan therapy in a placebo-controlled, cross-over design [57]. Several ongoing randomized trials are investigating the efficacy of sequential PAH-specific combination therapy, and are including PAH-CHD patients with closed defects, such as Beraprost Added to Inhaled Treprostinil (BEAT, clinicaltrials.gov NCT01908699), Early Combination of Oral Treprostinil with Background Oral Monotherapy (FREEDOM-Ev, clinicaltrials.gov NCT01560624), Pulsed, Inhaled NO added to Background Therapy in Symptomatic Patients (INovation-1, clinicaltrials.gov NCT02725372), Riociguat Replacing PDE-5i Therapy Evaluated Against Continued PDE-5i Therapy (REPLACE, clinicaltrials.gov NCT02891850), and a trial comparing triple therapy to dual therapy (treprostinil added to background ambrisentan and tadalafil, clinicaltrials.gov NCT02999906) (Table 3).

Upfront combination therapy, i.e., initiating with two or more PAH-specific therapies in treatment-naïve patients, is becoming increasingly commonplace in clinical practice. First-Line Ambrisentan and Tadalafil Combination Therapy in Subjects With Pulmonary Arterial Hypertension (AMBITION) was the first large-scale trial with 500 PAH patients to demonstrate that upfront combination therapy with ambrisentan and tadalafil resulted in a significantly lower risk for clinical failure events compared with monotherapy with either of these drugs [53]. In a pilot study of 17 patients with severe PAH, triple upfront combination therapy with intravenous epoprostenol, bosentan, and sildenafil improved hemodynamics, exercise capacity and WHO functional class, with a remarkable three-year survival rate of 100% compared to an otherwise expected three-year survival rate of 49% [82]. Several ongoing interventional studies which are also including patients with closed defects are investigating optimal upfront combination treatment strategies for PAH patients. The Prospective, Multicenter, Open-label Study Evaluating the Effects of First-line Oral Combination Therapy of Macitentan and Tadalafil in Patients With Newly Diagnosed Pulmonary Arterial Hypertension (OPTIMA) trial aims to evaluate upfront duo combination therapy of macitentan and tadalafil in an open-label, prospective design (clinicaltrials.gov NCT02968901). The Efficacy and Safety of Initial Triple Versus Initial Dual Oral Combination Therapy in Patients With Newly Diagnosed Pulmonary Arterial Hypertension (TRITON) trial is assessing the efficacy and safety of upfront triple combination therapy with macitentan, tadalafil, and selexipag compared with macitentan, tadalafil, and placebo (clinicaltrials.gov NCT02558231)

More research is required to guide management decision as to whether upfront combination therapy is superior to sequential combination therapy, and if so, which combinations of drugs are preferred. Also, more data are urgently needed on the potential safety and efficacy of advanced therapies for PAH-CHD patients, beyond those with ES or closed defects.

## 7. Interventional Approaches

Recent advances in surgical and percutaneous techniques have enabled repair or palliation of most cardiac defects, with low periprocedural risks. Timing of repair is crucial in the avoidance of development of PAH, as early changes in the pulmonary vascular bed are likely reversible if the shunt is repaired. In general, patients with prevalent systemic-to-pulmonary shunts and normal PVR may safely undergo shunt closure. Hemodynamically insignificant shunts do not require closure. In case of raised PVR and mean PAP and predominantly systemic-to-pulmonary shunting, repair may be considered, whereas in case of predominantly pulmonary-to-systemic shunting repair of the defect is contraindicated as the shunt facilitates RV decompression.

### 7.1. In Case of Moderately Elevated PVR

Clinical decision making for patients with borderline hemodynamics is more difficult and expert consensus guidelines propose remarkably different criteria for shunt closure [13,15,83] (Table 4). It is reasonable to recommend the so-called treat-and-repair strategy—neoadjuvant pulmonary vasodilator therapy with secondary repair of the shunt when PVR drops to acceptable levels. Theoretically, a good hemodynamic response to PAH-specific therapy might be indicative of reversibility and secondary repair of the shunt may help to prevent further deterioration to PAH. Case reports have described initial good outcomes [84,85,86], though the long-term consequences remain unclear. Recently, partial closure of septal defects (i.e., use of flap patch or fenestrated occluding device) has been reported [87,88] but recommendations defining optimal fenestration size await further investigation. No definitive data are available on the usefulness of vasoreactivity testing for operability assessment. Decisions for closure in these cases should be based on careful individual evaluation.

### 7.2. Decision to Intervene: To Close or Not to Close

Of note, a significant proportion of CHD patients who have undergone seemingly successful repair during childhood may develop PAH years after closure—estimated 15% lifetime risk of those with closed defects in the Dutch CONgenital CORvitia registry [89]. Accordingly, the decision to intervene should not be solely based on procedural and technical feasibility, but rather take into account the long-term prospects. A retrospective analysis identified that at baseline values of PVR ≥ 5 Wood units (WU), PVR index ≥ 6 WU·m^2^ and PVR/systemic vascular resistance ratio ≥ 0.33 are common in these patients [90], though it did not mention the number of patients that did not develop PAH despite similar values. Ongoing registries are anticipated to clarify strategies for identifying those at high risk for developing PAH after repair (clinicaltrials.gov NCT01659411).

In ES patients, the shunt should never be closed, as it may act as a “relief” valve for the excess pressure in the RV. In fact, the principle of a “relief” valve has been successfully used in idiopathic PAH patients with severe or drug-refractory PAH, by atrial septosomy [91] or creation of Potts shunt [92,93], a side-by-side anastomosis of the left pulmonary artery to the descending aorta.

### 7.3. Reversibility

Reversibility of PAH remains difficult to predict and data on markers for reversibility are scarce, though urgently required for the purpose of assessing operability. So far, the number of circulating endothelial cells might be a promising candidate as it was shown to be increased in CHD patients with irreversible PAH compared with those with reversible PAH [94]. Animal models have demonstrated several other promising targets to reliably identify the potential reverse remodeling, such as inhibition of phosphodiesterase-1 [95], activation of soluble guanylate cyclase [96], and dichloroacetate [97].

### 7.4. (Lost-to-)Follow-Up after Repair

Unfortunately, many CHD patients have been lost-to-follow-up or discharged after repair [98,99] and current guidelines state that regular follow-up is not required in those with repair under the age of 25 without relevant complications or residuae [15]. Nevertheless, “successful” repair—of even simple defects like ASD—with reassuring short-term outcomes is not invariably equivalent to favorable long-term outcome. Considering this alarming fact, we urge that CHD patients irrespective of age or success of repair are referred back to a tertiary care center for an appropriate assessment to determine if early pulmonary arterial remodeling is present, and recommend surveillance of all CHD patients with at least sporadic follow-up (i.e., once every five years).

## 8. Future Implications

### 8.1. Risk Stratification

Risk stratification is becoming increasingly important for refining prognostication of disease progression, treatment response and timing of intervention. In recent years, numerous predictors of mortality in PAH (e.g., 6-min walk distance, WHO functional class, maximum oxygen uptake, RV dysfunction, hemodynamic parameters, and biomarkers) have been identified and implemented in PAH guidelines for risk stratification and guidance of treatment. Given the distinct differences between PAH-CHD and other PAH etiologies, a modification of the widely used ESC/ERS guidelines table on disease severity, stability and prognostic parameters to PAH-CHD patients is necessary and one has been proposed by Gatzoulis et al. for ES patients [100], though its predictive value has not been validated. Data suggest that functional class, RV failure, arrhythmias, NT-proBNP, younger age at presentation and complex cardiac anatomy predict poor outcome in PAH-CHD [17,101,102,103].

### 8.2. Challenging Patient Groups: Down Syndrome and Fontan

Management of some CHD subgroups with elevated PVR is particularly challenging. Patients with Down syndrome are more prone to develop PAH earlier [104]. In fact, neonates with Down syndrome already have a higher incidence of pulmonary hypertension (1.2%–5.2%) compared to the general population (0.1%) [105]. Further, patients with Down syndrome tend to have a high prevalence of complex CHD—most commonly VSD and AVSD—associated with ES and worse functional outcome. Uncontrolled studies note similar favorable effects of PAH-specific treatment in ES patients with and without Down syndrome [63,106,107]. However, data from a nationwide registry showed that patients with Down syndrome received significantly less PAH-specific treatment [8].

Another challenging subgroup is the Fontan population—who strictly speaking does not meet the diagnostic criteria for PAH but may suffer from elevated PVR. As their pulmonary circulation is passively driven in the absence of a subpulmonary ventricle, even a mild increase in PVR may limit pulmonary blood flow and thus cardiac output. The introduction of advanced therapies in this population is an area of active investigation. Bosentan for six months had no favorable effects on exercise capacity, NT-proBNP levels, or quality-of-life in a randomized trial [108], whereas in a placebo-controlled trial bosentan improved exercise capacity, exercise time, and functional class in adolescents and adults without causing serious adverse events [109]. Another RCT showed that sildenafil improved exercise capacity in children and adolescents [110]. Further investigation is needed before therapeutic recommendations in this patient population can be made.

### 8.3. New Candidate Therapies in PAH

Ongoing studies are addressing whether there is a role for classic heart failure therapies in the treatment of PAH. Spironolactone is being evaluated in two RCTs, as monotherapy or sequential therapy to ambrisentan (clinicaltrials.gov, NCT01712620, NCT02253394). Another trial is assessing the effect of adding beta-blockers to background therapy (clinicaltrials.gov, NCT02507011).

The current PAH-specific drugs are able to slow disease progression, but cannot be considered a cure. Although over the last decades many drugs belonging to the same classes have been developed, we perhaps should consider pursuing the development of truly novel therapies—that could potentially reverse or cure PAH—rather than reinvesting in the same classes of drugs or their combinations. There is no shortage of such new candidate therapies for PAH that target alternative pathobiological pathways like metabolic or inflammatory approaches or explore new treatment strategies like regenerative medicine. l-citrulline, a NO-independent stimulator, was evaluated in 20 idiopathic PAH and five ES patients, and showed significant improvement in exercise capacity, hemodynamics, and quality-of-life after two weeks [111]. Imatinib, a selective antagonist of the platelet-derived growth factor receptor, seemed effective in patients with severe or drug-refractory PAH [112,113], but in a larger trial serious adverse events and study drug discontinuations were common—making the clinical implications of this drug questionable. New candidate drugs with ongoing or planned studies include the competitive, reversible protease inhibitor ubenimex (clinicaltrials.gov NCT02736149), oral selective IP receptor agonist ralinepag (clinicaltrials.gov NCT02279160), semi-synthetic triterpenoid bardoxolone methyl (clinicaltrials.gov NCT02036970), inhaled prostacyclin receptor agonist QCC374 (clinicaltrials.gov NCT02927366), and autologous progenitor cell-based gene therapy (clinicaltrials.gov NCT00469027) (Table 3).

Some of the limitations discussed in the prior randomized trials, such as including one subgroup of PAH-CHD (i.e., patients with PAH after defect closure) and limited power for subgroup analyses, are applicable to these ongoing trials. Still, these trials with new candidate drugs may potentially transform the therapeutic approach of PAH-CHD.

## 9. Conclusions

Identifying the optimal treatment for the heterogeneous population with PAH-CHD demands a thorough and expert approach. Over the past years, considerable progress has been made in the field of PAH with the introduction of advanced PAH-specific therapies. Cumulative evidence now supports the introduction of these therapies in PAH-CHD patients including patients with ES or closed defects—though less research has focused on other types of PAH-CHD. Although many challenges regarding the clinical management of PAH-CHD still remain, ongoing studies are likely to complement current insights and some may revolutionize the treatment of PAH in CHD. Research on risk stratification and the impact of genetic background may further enhance our understanding of the pathophysiology and help to determine the best therapeutic and management approaches. These aforesaid developments are bound to improve quality-of-life and survival prospects for PAH-CHD patients.

## Figures and Tables

**Table 1:** Clinical classification of pulmonary arterial hypertension associated with congenital heart disease.

Subgroup	Clinical Classification
(i)	**Eisenmenger syndrome** Includes intra- and extracardiac defects with initial systemic-to-pulmonary shunts leading to elevated PVR with ultimate reversal or bidirectional shunting and development of central cyanosis at rest.
(ii)	**PAH associated with prevalent systemic-to-pulmonary shunt lesions** PVR is mild to moderately elevated in presence of moderate to large systemic-to-pulmonary shunt, either correctable or not. No cyanosis at rest.
(iii)	**PAH with small or coincidental cardiac defects** The small cardiac defect (usually ASD < 2 cm and VSD < 1 cm of effective diameter assessed by echocardiography) is generally considered coincidental and unrelated to the marked elevation in PVR. The clinical picture is very similar to idiopathic PAH.
(iv)	**PAH after defect closure** PAH is either persisting or recurring immediately or months to years after closure of the cardiac defect in the absence of relevant postoperative hemodynamic lesions.

Note: ASD = atrial septal defect; PVR = pulmonary vascular resistance; VSD = ventricular septal defect. Modified from Galiè et al. [13].

**Table 2:** Summary of double-blind, randomized, placebo-controlled trials.

First Author (Study Acronym)	*n* with CHD (% of Study Population)	Background Therapy	Intervention	Follow-Up (Weeks)	Primary Outcome	Study Conclusion
**Study Population Including Mixed Group of PAH-CHD**						
Simonneau et al. [42]	109 (23)	None	Treprostinil	12	Δ 6MWD	↑ PVR, symptoms
Galiè et al. [43] (EARLY)	32 (17)	None	Bosentan	26	Δ 6MWD and PVR	↑
Galiè et al. [44] (PHIRST)	47 (12)	None or bosentan	Tadalafil	16	Δ 6MWD	↑ TTCW
Ghofrani et al. [45] (PATENT-1)	35 (8)	None, ERA or prostanoids	Riociguat	12	Δ 6MWD	↑ PVR, WHO, TTCW, NT-proBNP
**Study Population Including PAH with Closed Defects**						
Simonneau et al. [46] (PACES)	10 (4)	Epoprostenol	Sildenafil	16	Δ 6MWD	↑ PVR, TTCW, QoL
Galiè et al. [47] (SUPER-1)	18 (6)	None	Sildenafil	12	Δ 6MWD	↑ WHO class, and hemodynamics
Tapson et al. [48] (FREEDOM-C)	22 (6)	ERA, PDE-5i or both	Treprostinil	16	Δ 6MWD	=
Tapson et al. [49] (FREEDOM-C2)	4 (1)	ERA, PDE-5i or both	Treprostinil	16	Δ 6MWD	=
Pulido et al. [50] (SERAPHIN)	62 (8)	PDE-5i or prostanoids	Macitentan	85–104	TTCW	↑
Jing et al. [51] (FREEDOM-M)	18 (5)	None	Treprostinil	12	Δ 6MWD	↑
McLaughlin et al. [52] (COMPASS-2)	20 (6)	Sildenafil	Bosentan	+/− 170	TTCW	=
Galiè et al. [53] (AMBITION)	13 (3)	None	Ambrisentan + tadalafil vs. ambrisentan vs. tadalafil	74	TTCW	↑ with initial combination therapy > ambrisentan or tadalafil monotherapy
Sitbon et al. [54] (GRIPHON)	110 (10)	None, ERA, PDE-5i or both	Selexipag	67	TTCW	↑ in all baseline treatment groups
**Study Population Including Eisenmenger Syndrome**						
Galiè et al. [55] (BREATHE-5)	54 (100)	None	Bosentan	16	Δ Spo2 and PVR	↑ without compromising SpO2
Singh et al. [56]	10 (50)	None	Sildenafil	6	Δ 6MWD	↑ NYHA class, PVR
Iversen et al. [57]	21 (100)	None	Bosentan, add sildenafil cross-over	39	Δ 6MWD	Bosentan alone ↑ with addition of sildenafil =, but ↑ SO2
Mukhopadhyay et al. [58]	28 (100)	None	Tadalafil	6	Δ 6MWD	↑ WHO, SO2, and PVR

Note: Randomized controlled trials with beraprost and sitaxentan were not included in this table, as they have not been approved for treatment of pulmonary arterial hypertension in Western countries or have been withdrawn. Δ = change; ↑ = improved; = = no statistically significant difference; 6MWD = 6-min walk distance; AMBITION = Ambrisentan and Tadalafil in Patients with Pulmonary Arterial Hypertension; BREATHE-5 = Bosentan Randomized Trial of Endothelin Antagonist Therapy-5; CHD = congenital heart disease; COMPASS-2 = Effects of the Combination of Bosentan and Sildenafil versus Sildenafil Monotherapy on Pulmonary Arterial Hypertension; EARLY = Endothelin Antagonist Trial in Mildly Symptomatic Pulmonary Arterial Hypertension Patients; ERA = endothelin-receptor antagonist; FREEDOM-C = Oral Treprostinil in Combination With an Endothelin Receptor Antagonist and/or a Phosphodiesterase-5 Inhibitor for the Treatment of Pulmonary Arterial Hypertension; FREEDOM-C2 = Oral Treprostinil for the Treatment of Pulmonary Arterial Hypertension in Patients Receiving Background Endothelin Receptor Antagonist and Phosphodiesterase-5 Inhibitor Therapy; FREEDOM-M = Oral Treprostinil as Monotherapy for the Treatment of Pulmonary Arterial Hypertension; GRIPHON = Prostacyclin Receptor Agonist In Pulmonary Arterial Hypertension Study; NT-proBNP = n-terminal pro brain natriuretic peptide; NYHA = New York Heart Association; PACES = Pulmonary Arterial Hypertension Combination Study of Epoprostenol and Sildenafil; PAH-CHD = pulmonary arterial hypertension associated with congenital heart disease; PATENT-1 = Pulmonary Arterial Hypertension Soluble Guanylate Cyclase–Stimulator Trial 1; PDE-5i = phosphodiesterase-5 inhibitor; PHIRST = Pulmonary Arterial Hypertension and Response to Tadalafil; QoL = quality-of-life; SERAPHIN = Study with an Endothelin Receptor Antagonist in Pulmonary Arterial Hypertension to Improve Clinical Outcome; SO2 = systemic oxygen saturation; SpO2 = peripheral capillary oxygen saturation; SUPER-1 = Sildenafil Use in Pulmonary Arterial Hypertension; WHO = World Health~Organization.

**Table 3:** Summary of ongoing randomized, placebo-controlled trials.

Study (Clinicaltrial.gov)	Phase	Patients	Intervention	Primary Outcome	Anticipated Completion
**Combination Therapy with Currently Available PAH-Specific Therapy**					
BEAT (NCT01908699)	III	PAH on inhaled treprostinil	Beraprost vs. placebo	TTCW	2017
TRITON (NCT02558231)	III	PAH diagnosis <6 months	Macitentan + tadalafil + selexipag vs. macitentan + tadalafil + placebo	PVR	2018
FREEDOM-Ev (NCT01560624)	III	PAH on monotherapy	Oral treprostinil vs. placebo	TTCW	2018
INOvation-1 (NCT02725372)	III	PAH on therapy	Inhaled NO vs. placebo	Δ 6MWD	2018
REPLACE (NCT02891850)	IV	PAH on PDE-5i	Switch to riociguat vs. standard-of-care	Δ 6MWD, WHO class, NT-proBNP	2018
Triple vs. dual therapy (NCT02999906)	III	PAH on ambrisentan + tadalafil	Treprostinil vs. placebo	Δ 6MWD	2022
**Heart Failure Therapy**					
CAPS-PAH (NCT02253394)	IV	PAH on ambrisentan	Spironolactone vs. placebo	Δ 6MWD, pVO2	2017
Spironolactone (NCT01712620)	II	PAH	Spironolactone vs. placebo	Δ 6MWD, TTCW	2018
Beta-blockers (NCT02507011)	II	PAH on therapy	Carvedilol vs. placebo	Δ RVEF	2018
**New Candidate Drugs**					
LIBERTY (NCT02664558)	II	PAH, on therapy	Ubenimex vs. placebo	PVR	2017
APD811 in PAH (NCT02279160)	II	PAH, on therapy	Ralinepag vs. placebo	PVR, Δ 6MWD	2017
LARIAT (NCT02036970)	II	PAH	Bardoxolone methyl vs. placebo	Δ 6MWD	2018
QCC374 in PAH (NCT02927366)	II	PAH on therapy	QCC374 vs. placebo	PVR	2019
SAPPHIRE (NCT03001414)	II	PAH on therapy	Autologous progenitor cell-based gene therapy vs. placebo	Δ 6MWD	2020

Note: Δ = change; ES = Eisenmenger syndrome; pVO2 = peak oxygen consumption; RVEF = right ventricular ejection fraction; BEAT = Beraprost Added to Inhaled Treprostinil; FREEDOM-Ev = Early Combination of Oral Treprostinil with Background Oral Monotherapy; INOvation-1 = Pulsed, Inhaled NO added to Background Therapy in Symptomatic Patients; LARIAT = Bardoxolone Methyl Evaluation in Patients With Pulmonary Hypertension; LIBERTY = A Study of Ubenimex in Patients With Pulmonary Arterial Hypertension; REPLACE~=~Riociguat Replacing PDE-5i Therapy Evaluated Against Continued PDE-5i Therapy; CAPS-PAH = The Combination Ambrisentan Plus Spironolactone in Pulmonary Arterial Hypertension Study; SAPPHIRE = Study of Angiogenic Cell Therapy for Progressive Pulmonary Hypertension: Intervention with Repeat Dosing of Endothelial NO-synthase-enhanced Endothelial Progenitor Cells; TRITON = The Efficacy and Safety of Initial Triple Versus Initial Dual Oral Combination Therapy in Patients With Newly Diagnosed Pulmonary Arterial Hypertension.

**Table 4:** Guideline recommendations for correction in congenital heart disease with prevalent systemic-to-pulmonary shunts.

Type	Correctable?	AHA/ACC CHD Guidelines, 2008 [83]	ESC GUCH Guidelines, 2010 [15]	ESC/ERS PH Guidelines, 2015 [13] *
ASD	Yes	All with RA and RV enlargement with or without symptoms	RV volume overload and PVR < 5 WU regardless of symptoms	PVRi < 4 WU·m^2^ or PVR < 2.3 WU
	No	Severe irreversible PAH and no L-R shunt	ES	PVRi > 8 WU·m^2^ or PVR > 4.6 WU
	Individual patient evaluation	Paradoxical embolism or orthodeoxia-platypnea Net L-R shunt, PVR < 2/3 of SVR, PAP < 2/3 of systemic levels or when responsive to pulmonary vasodilators or test occlusion of defect	Paradoxical embolism PVR ≥5 WU but <2/3 of SVR or PAP < 2/3 of systemic levels and net L-R shunt (Qp:Qs > 1.5)	PVRi 4–8 WU·m^2^ or PVR 2.3–4.6 WU
VSD	Yes	Qp:Qs ≥ 2 and LV volume overload History of IE	Symptoms of L-R shunting and no severe PVD History of IE Asymptomatic with LV volume overload due to VSD	PVRi < 4 WU·m^2^ or PVR < 2.3 WU
	No	Severe irreversible PAH	ES or exercise induced desaturation VSD is small, not subarterial and no LV volume overload/PH	PVRi > 8 WU·m^2^ or PVR > 4.6 WU
	Individual patient evaluation	Net L-R shunt (Qp:Qs > 1.5) and PAP < 2/3 of SVR and PVR < 2/3 of systemic levels Net L-R shunt (Qp:Qs > 1.5) and LV systolic/diastolic failure	Net L-R shunt (Qp:Qs > 1.5) and PAP or PVR < 2/3 of systemic levels	PVRi 4–8 WU·m^2^ or PVR 2.3–4.6 WU

* In the ESC/ERS guidelines for the diagnosis and treatment of PH, there is no distinction between ASD and VSD closure. AHA/ACC = American Heart Association/American College of Cardiology; ESC = European Society of Cardiology; ERS = European Respiratory Society; GUCH = grown-ups with congenital heart disease; IE = infective endocarditis; L-R = left-to-right (systemic-to-pulmonary); PAP = pulmonary arterial pressure; PVD = pulmonary vascular disease; PVRi = pulmonary vascular resistance index; Qp:Qs = the ratio of pulmonary blood flow to systemic blood flow; SVR = systemic vascular resistance; WU = Woods Units.
